# Socioeconomic Deprivation and Dropout from Contemporary Psychological Intervention for Common Mental Disorders: A Systematic Review

**DOI:** 10.1007/s10488-021-01178-8

**Published:** 2021-11-27

**Authors:** Nick Firth, Michael Barkham, Jaime Delgadillo, Kai Allery, Jonathan Woodward, Alicia O’Cathain

**Affiliations:** 1 School of Health and Related Research, University of Sheffield, Sheffield, S10 2TN UK; 2 Clinical and Applied Psychology Unit, University of Sheffield, Sheffield, S10 2TN UK; 3 Medical School, University of Sheffield, Sheffield, S10 2TN UK

**Keywords:** Dropout, Deprivation, Socioeconomic, Common mental disorders, Review, Meta-analysis

## Abstract

Dropout during psychological intervention is a significant problem. Previous evidence for associations with socioeconomic deprivation is mixed. This study aimed to review the evidence for associations between deprivation and dropout from contemporary adult psychological interventions for common mental disorders (CMDs). Systematic review, narrative synthesis and random effects meta-analysis of peer-reviewed English language journal articles published June 2010–June 2020 was conducted.﻿ Data sources included medline, PsycInfo, databases indexed by web of science, ProQuest social science database and sociology collection, and the Cochrane Library, supplemented by forward and backward citation searching. Five studies were eligible for inclusion (mean *N* = 170, 68% female, 60% White Caucasian, 32% dropout rate, predominantly cognitive behaviour therapy/cognitive processing therapy). Narrative synthesis indicated an overall non-significant effect of deprivation on dropout. Meta-analytic significance of controlled (*k* = 3) and uncontrolled (*k* = 4) effects depended on the measure of deprivation included for those studies using more than one measure (controlled OR 1.21–1.32, p = 0.019–0.172, uncontrolled OR 1.28–1.76, p = 0.024–0.423). The low number of included studies meant sub-group comparisons were limited, despite some tentative indications of potential differential effects. A comparator set of excluded studies showed similar uncertainty. There was limited evidence that did not overall suggest a clear significant effect of deprivation on dropout from contemporary individual CMD interventions. However, more contemporary research is needed, as effects may vary according to clinical and methodological factors, and for dropout versus non-initiation.

**Supplementary Information:**

The online version contains supplementary material available at 10.1007/s10488-021-01178-8.

## Introduction

There is a consistent, well-documented evidence base linking socioeconomic deprivation with a broad range of problems and inequalities (Cookson et al., 2018) across social, physical, and mental health domains (Delgadillo et al., 2016; Eibner et al., 2004; Kuruvilla & Jacob, 2007; O'Donoghue et al., 2016).

Socioeconomic deprivation typically refers to a lack of social and/or economic resources important to living quality (Poverty & Social Exclusion UK, 2016; Townsend, 1979, 1987). Socioeconomic *status*, arguably a subtype of deprivation, has most typically been measured by a combination of income, education, and occupation, either on an individual or household level (American Psychological Association, 2007). However, socioeconomic deprivation may also act at other levels such as a person’s neighbourhood, and may be measured using metrics such as crime, housing, local services, living environment, and health in addition to those described above (Ministry of Housing‚ Communities & Local Government, 2019).

In addition to evidence linking deprivation with greater prevalence of common mental disorders (CMDs) and associated referrals (Delgadillo et al., 2018; Fryers et al., 2003; O'Donoghue et al., 2016), inequalities in the receipt of mental health care have also been found. People living in areas of greater deprivation may be less likely to receive psychological intervention (for example via reduced availability or uptake) (Berzins et al., 2018; Delgadillo et al., 2016, 2018; Grant et al., 2012), and the intervention they receive may be less effective on average (Finegan et al., 2018, 2020). This is consistent with the concept of the inverse care law (Hart, 1971), such that the availability of good health care is inversely proportional to the need of the population.

In recent years, efforts have focused on improving access to treatment and reducing care inequalities for people with CMDs, such as the national improving access to psychological therapies (IAPT) programme in England (Clark et al., 2018; NHS Digital, 2020; The National Collaborating Centre for Mental Health, 2019). Despite this, associations between intervention outcomes and deprivation continues to be demonstrated—all significant effects in Finegan et al.’s (2018) recent meta-analysis were published in the last ten years.

This suggests potential ongoing inequalities throughout CMD care pathways that may be limiting clinical effectiveness. Unilateral discontinuation of treatment (also known as treatment dropout) is one factor known to be associated with reduced clinical effectiveness (Barrett et al., 2008; Cahill et al., 2003; Zieve et al., 2019). The success of costly initiatives to directly improve treatment effectiveness (for example, targeted at patients experiencing deprivation) may be limited if such patients do not remain in treatment long enough to experience them. In contrast, understanding dropout could not only improve theoretical models, but also provide a specific “upstream” focus for interventions to enable patients to find more benefit from treatment.

Dropout can also impact clinically and financially on treatment delivery at an organisational level, for example via increased waiting times, detrimental outcomes for other patients, waste of financial and human resources, staff morale/turnover, and negative community perception (Barrett et al., 2008; Klein et al., 2003; Moore et al., 2001). Rates of dropout from psychological treatment are typically estimated at 20–35% (Cooper & Conklin, 2015; Roos & Werbart, 2013; Swift & Greenberg, 2012).

Previous reviews and meta-analyses examining the association between deprivation and dropout from psychological therapy have indicated certain significant associations between deprivation and dropout (Baekeland & Lundwall, 1975; Swift & Greenberg, 2012; Wierzbicki & Pekarik, 1993). However, early reviews used non-systematic search strategies. They have typically focused on a broad range of predictors, preventing a detailed critical review of socioeconomic deprivation. They have typically included only individual-level socioeconomic variables (excluding e.g., neighbourhood level predictors; Finegan et al., 2020; O’Donoghue et al., 2016; Richardson et al., 2015). They have focused on mental health conditions in general, making conclusions specifically about CMDs difficult, particularly given evidence of differential dropout rates for people with different diagnoses (Swift & Greenberg, 2012). They have also tended to focus solely on traditional psychotherapy, often using very narrow and limited search terms and therefore potentially excluding relevant studies of psychological intervention.

Furthermore, in recent years the provision of psychological intervention has continued to evolve and change, including increased adoption of stepped care delivery systems, a focus on improving access to intervention, and increasing utilisation of telephone and computer-based interventions, in addition to peripheral technology such as text message communication/reminders (Davison, 2000; Department of Health, 2008, 2011). These approaches may reduce barriers to treatment completion for certain groups—for example, by reducing transport costs, or allowing parents who do not have access to alternative childcare to stay at home with younger children. Conversely, they may bring additional challenges—for example, staying at home may reinforce social isolation, whilst computer interventions may rely more on patients’ digital/literacy skills. As such, there is a need for an update of the evidence on deprivation and dropout that reflects the contemporary psychological intervention context.

Finally, this review focuses specifically on dropout once treatment has begun, as distinct from treatment non-initiation. In addition to theoretical arguments for this distinction (e.g., Garfield, 1989), empirical evidence suggests that factors predicting dropout in-treatment may differ from those predicting non-initiation (Kehle-Forbes et al., 2016; Kline et al., 2020; Miller et al., 2019). Kline et al. (2020) suggest that nuance and specificity are likely to be lost if non-initiation and dropout during treatment are not considered as separate heterogeneous types of discontinuation.

### Aims

This review aimed to assess the evidence for associations between socioeconomic deprivation and dropout from contemporary psychological interventions for adults with common mental disorders, by systematically reviewing evidence from peer-reviewed published journal articles. The review question was: To what extent (and in which contexts) is socioeconomic deprivation associated with dropout from contemporary psychological intervention?

The review aimed to focus in particular on evidence regarding indicators of socioeconomic deprivation at the patient level versus neighbourhood level, as well as the impact in different intervention delivery modalities (e.g., face-to-face, telephone, computer-based). The review aimed to minimise heterogeneity risked by the often broad and unfocused inclusion criteria used in previous reviews, by using a refined and targeted search strategy to focus on a specific phenomenon (contemporary in-treatment dropout) in a specific population (people experiencing CMDs).

## Methods

Details of this review including the protocol were pre-registered on the PROSPERO International prospective register of systematic reviews (registration number 187034; Firth et al., 2020).

### Study Eligibility

A PICO(SS) framework is presented in Table 1 to summarise the population, intervention, comparator, outcomes, setting, and study design inclusion criteria. The review focused on individually delivered psychological interventions. This decision was made in part to improve homogeneity—in particular, dropout from group and couple treatments may be influenced by other treatment attenders (Firth et al., 2019). Psychological interventions were allowed to be supplemented by other interventions (e.g., pharmacological), as long as the psychological intervention was the primary component of treatment, acknowledging CMD treatment guidelines (National Institute for Health and Care Excellence, 2009, 2011b).

**Table 1:** PICOSS framework

	Eligibility criteria	Exclusions
Population	Adults aged 18 or over who received an individually-delivered psychological intervention for a common mental disorder	People aged 17 or under
Intervention	Individually delivered outpatient psychological intervention designed primarily to treat at least one common mental disorder, using any modality (e.g., 1:1 face-to-face, telephone, or computerized interventions)	Group or couples interventions, non-psychological interventions, interventions not focused on treating a common mental disorder
Comparator	Within-group comparison between patients experiencing different extents of socioeconomic deprivation, as assessed by relevant measures of socioeconomic deprivation	
Outcomes	Measures of dropout from intervention	
Setting	Any outpatient setting delivering psychological interventions, worldwide	Inpatient settings, penal settings, etc
Study	Peer-reviewed and published empirical quantitative studies reported in English between June 2010 and June 2020	Qualitative studies, theoretical papers, etc

Interventions were required to focus primarily on one or more CMDs. CMDs were first described as “disorders which are commonly encountered in community settings, and whose occurrence signals a breakdown in normal functioning” (Goldberg & Huxley, 1992, pp. 7–8). Despite challenges and variation in classification (Goldberg & Huxley, 1992), for this review, CMDs were considered to include: depressive disorders (excluding bipolar disorder), anxiety disorders (including panic and phobic disorders, obsessive–compulsive disorder and body dysmorphic disorder), and post-traumatic stress disorder (PTSD), in line with UK national guidance (National Institute for Health and Care Excellence, 2011a). Examples of exclusions include alcohol and substance use disorders, psychosis and schizophrenia.

Studies were required to include a measure of socioeconomic deprivation as a comparator. Socioeconomic deprivation is defined in this review as the extent of relative disadvantage or lack of resources that contribute to standards of living (e.g., social and economic/material resources) (Bartley & Blane, 1994; Poverty & Social Exclusion UK, 2016; Townsend, 1979, 1987).

The outcome measure for this review was dropout from the psychological intervention. Dropout was defined as occurring when a patient who has begun intervention (i.e. attended at least one session) then ends treatment before reaching “mutual agreement that therapy has been completed” (Garfield, 1989). This excluded studies defining dropout as including patients who did not attend any sessions—consistent with Garfield (1989) and empirical literature (Kehle-Forbes et al., 2016; Kline et al., 2020; Miller et al., 2019), this review considers those patients as rejecting or failing to initiate treatment, rather than dropping out.

Common operationalisations of dropout include those based on *treatment duration, therapist judgement,* and/or *termination by failure* (discharge due to failing to attend)*.* Duration-based definitions have been widely challenged and it has been suggested that they be treated with caution (Brandt, 1965; Fiester et al., 1974; Pekarik, 1985; Wierzbicki & Pekarik, 1993). Duration-based measures were only included where there was a consistent agreed treatment duration (e.g., the intervention was pre-specified as being 8 sessions but the patient only attended up to session 5). We believe that to do otherwise risks misrepresenting mutually agreed briefer interventions. Termination by failure is a relatively conservative approach, and therapist judgement has been recommended over other approaches (Wierzbicki & Pekarik, 1993). Therapist judgement can incorporate other operationalisations (e.g., a therapist can decide whether or not the patient has attended for an appropriate duration of treatment, or capture dropout via failure to attend when appropriate). It also arguably has face validity—typical definitions of dropout require a therapist decision in one form or another, although there is also arguably an increased risk of subjective variability in dropout decisions. In order to best represent the available evidence, different operationalisations of dropout were permitted, as long as they captured some measure of unilateral termination after at least one session had been attended, and before a specified treatment completion criterion was met. Rate of dropout and significance of predictors have at times been found to be associated with the measurement used by the study (Pekarik, 1985; Wierzbicki & Pekarik, 1993). As such, dropout definition was planned as a meta-analysis moderator and subgroup for narrative analysis.

This review included papers published in the ten-year period from June 2010 to June 2020. This decision was influenced by recent changes in contemporary psychological intervention delivery and expansion in thinking around inequality and access (e.g., Delgadillo et al., 2018; Johansson & Andersson, 2012; Wakefield et al., 2020), and to align with Swift and Greenberg’s meta-analysis (2012), which included articles up to June 2010. Focusing on contemporary interventions was a key aspect of this review’s design.

### Systematic Search and Selection Process

A systematic electronic database search was supplemented by backward and forward citation searching for eligible studies (Cooper et al., 2018). Databases are listed in Table 2 (Booth, 2016; Gusenbauer & Haddaway, 2020). Searches were completed by 28 July 2020. Assessment of studies for inclusion was independently performed by NF and KA/JW and cross checked at each stage.

**Table 2:** Databases and search platforms

Platform	Database(s)	Search type
Ovid	Medline	Subject heading and text search
Ovid	PsycInfo	Subject heading and text search
Web of science	Web of science core collection, BIOSIS citation index, BIOSIS previews, Data citation index, KCI-Korean journal database, Russian science citation index, SciELO citation index	Text search
ProQuest	Social science database, sociology collection	Text search
Cochrane library	Cochrane central register of controlled trials	Text search

An example set of search terms is included in Supplementary Material. Generation of search terms was supplemented by collating terms from existing reviews. Reviews were identified by preliminary search [e.g., *“(dropout OR attrition OR *etc.*…) AND review)”*] and top reviews selected using impact, relevance, and recency rankings. Relevant search terms from these reviews were then added to the search strategy for this review.

### Data Extraction

Data was extracted by NF using forms (Supplementary Material) adapted from the Cochrane Collaboration data collection form for RCTs and non-RCTs (randomised controlled trials). JW independently verified 10% of extracted data items. Study authors were contacted where missing data was critical to interpretation of findings. Risk of bias/quality assessment was conducted using the Newcastle–Ottawa quality assessment scale (NOS; Wells et al. 2000) independently by NF and KA. Bias/quality assessments were used to interpret evidence a) on a per study basis, and b) as regards the overall quality of the literature base, and quality was planned as a meta-analysis moderator and subgroup for narrative analysis. For this purpose, study quality was recoded as a 5 level ordinal variable: very low (0–1), low (2–3), moderate (4–5), high (6–7), or very high (8–9) quality. Although funnel plots were planned to assess bias, the Cochrane Handbook advises that they should only be used where meta-analyses include at least 10 studies (Higgins et al., 2019).

### Data Synthesis

Data were narratively synthesised and meta-analysed. Dependent on included studies, planned narrative subgroup comparisons (and meta-analysis subgroups) were as follows: (a) delivery method (face-to-face, telephone, online, other), (b) measure of dropout (e.g., termination-by-failure vs. therapist judgement), (c) measure of deprivation (e.g., income vs. education, individual vs. neighbourhood level), (d) mental health disorder type (e.g., depressive disorders vs. anxiety disorders), and (e) study quality.

Effect sizes and 95% confidence intervals (CIs) were reported where available, and calculation attempted where unavailable. In this review “significance” refers to alpha = 0.05 (95%) two-tailed significance.

Random effects inverse variance meta-analyses were conducted using the metagen function in RStudio (Borenstein et al., 2010; Higgins et al., 2019). Heterogeneity was tested using Cochran’s Q and the I^2^ statistic. I^2^ was the primary heterogeneity test, given it is not biased by number of studies. The Cochrane Handbook provide a (rough) guide to interpreting I^2^: 0–40% may not be important; 30–60% may be moderate heterogeneity; 50–90% may be substantial heterogeneity; 75–100% considerable heterogeneity. In contrast, Cochran’s Q is underpowered for low numbers of studies, and overpowered for high numbers (Gavaghan et al., 2000; Higgins et al., 2003, 2019), so a p value of 0.10 was used as recommended (Higgins et al., 2019). Strategies recommended by the Cochrane Handbook to account for heterogeneity were explored as appropriate.

## Results

**Fig. 1:**
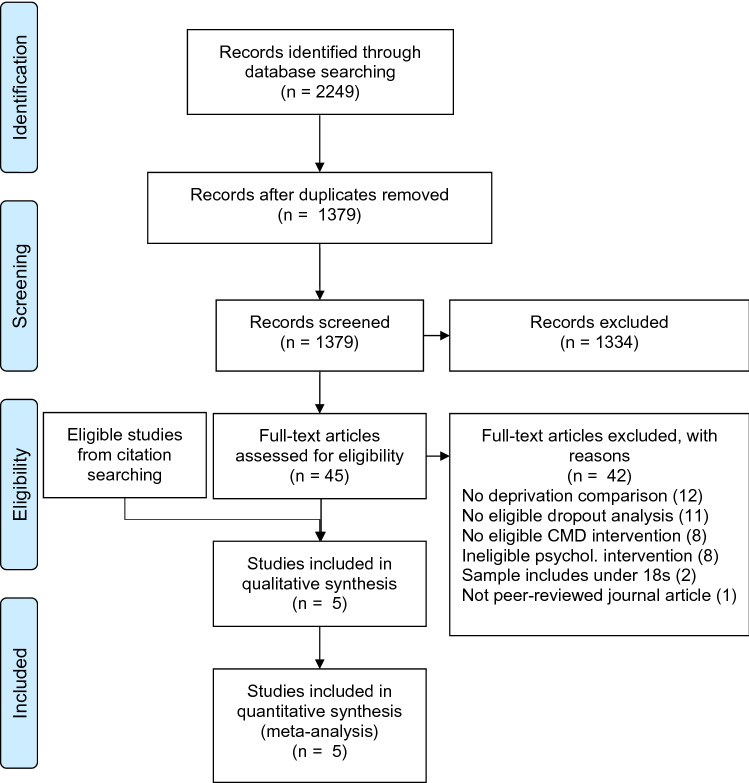
PRISMA flow diagram of included and excluded studies

There were 1,379 unique records screened (Fig. 1). There was 96.8% inter-rater agreement (Cohen’s kappa = 0.420; CI 0.28–0.56, indicating moderate agreement; Landis & Koch, 1977). Kappa is known to exhibit limitations, particularly where the agreement due to chance is skewed, as in this case. After reaching agreement, there were 45 full texts assessed for eligibility. Inter-rater agreement was 86.7% (Cohen’s kappa = 0.43; CI 0.07–0.80, moderate agreement). After reaching agreement, three studies were eligible for inclusion.

Backward and forward citation searching of eligible full text articles was performed—after removing duplicates, 166 records were screened. There was 97.6% inter-rater agreement (Cohen’s kappa = 0.83; CI 0.67—0.99, indicating almost perfect agreement). After reaching agreement, 14 full texts were assessed for eligibility. Inter-rater agreement was 86.7% (Cohen’s kappa = 0.60; CI 0.12—1.00, moderate agreement). After reaching agreement, 2 studies were eligible for inclusion, giving a total of five studies included in the review.

### Summary of Included Studies

An overview of study characteristics is shown in Table 3. Mean sample size was 170 participants (*SD* = 100; range 56 to 308). Mean dropout rate was 32% (*SD* = 10%; range 15–41%). Average age across studies was between 34 and 50 (one study reported median rather than mean age). Mean percentage of female participants was 68% (range 10–100%). White Caucasian participants were the racial/ethnic majority in three of four studies reporting this (mean 60%, range 32–79%). Employment status was only reported by one study (Mott et al., 2014; 46% employment).

**Table 3:** Summary characteristics of included studies

First author (Year)	Design	Target disorder	Population	Country	*N* completers/dropouts	Dropout %	Female %	Intervention
Binnie (2016)	Observational	CMDs	Primary care	England	140/61	30.3	63.4	CBT
Holder (2019)	RCT^a^	PTSD (military, sexual)	Veterans	US	33/23	41.1	100.0	CPT and written account
Lester (2010)	RCT^a^	PTSD (violence)	Female victims of violence	US	199/109	35.4	100.0	CPT/CT/PE/written account
Mott (2014)	Observational	PTSD	Veterans	US	58/33	36.3	8.8	CPT and/or PE
Schindler (2012)	Observational	Depression	University clinic	Germany	164/29	15.0	68.4	CBT

*CBT* cognitive behavioural therapy, *CPT* cognitive processing therapy, *CT* cognitive therapy, *CMDs* common mental disorders, *PE* prolonged exposure, *PTSD* post-traumatic stress disorder, *RCT* randomised controlled trial

^a^Secondary analysis of data from one or more randomised trials

All studies delivered interventions face-to-face (two did not explicitly specify, but are strongly inferred due to context). Whilst two studies used a fixed treatment duration (12–13 h; Holder et al., 2019; Lester et al., 2010), the remainder offered more variable treatment duration. There were no co-interventions specified by any study.

Eight deprivation variables were analysed across the five included studies (i.e., some studies used more than one measure of deprivation; Table 4). The majority of studies (four of five) used individual/household level measures of deprivation—these included educational level (four studies), income level (two studies), and employment status (one study). In contrast, Binnie and Boden (2016) used a neighbourhood level measure—the index of multiple deprivation (IMD; Department for Communities & Local Government, 2011). Measures of deprivation used by more than one study (i.e. education and income) were operationalised both categorically and continuously by different studies.

**Table 4:** Summary of deprivation variables used by included studies

First author (year)	Measure	Level	Operationalisation	Summary statistics
Binnie (2016)	IMD	Neighbourhood	Binary—Lower vs. higher deprivation than UK average	74% lower than UK average
Holder (2019)	Education	Individual	Continuous—years of education	Mean 14.4 years (*SD* = 2.03)
Lester (2010)	Education	Individual	Continuous—years of education	Mean 14.1 years (*SD* = 2.28–2.61)
Mott (2014)	Education	Individual	Binary—post high school educated or not	59.5% post high school educated
Schindler (2012)	Education	Individual	Binary—more than 12 years of education or not	44.0% more than 12 years
Lester (2010)	Income	Household	Categorical—6-point annual income scale	Mean $10,001–20,000
Mott (2014)	Income	Individual	Continuous—annual income	Mean $36,000 (*SD* = $27,000)
Mott (2014)	Employment	Individual	Binary—employed or not	46.2% employed

Some studies appear more than once due to analysing more than one measure of deprivation

*IMD* index of multiple deprivation

Regarding measurement of treatment completion/dropout, two studies used duration-based measures (Holder et al., 2019; Lester et al., 2010). Binnie and Boden (2016) used a therapist judgement definition. The final two studies used combinations of therapist judgement and duration-based measures (Mott et al., 2014; Schindler et al., 2013). Schindler et al. (2013) also excluded drop-outs for neutral reasons (such as moving out of area), to produce an outcome they term “quality-associated dropout”.

### Quality and Risk of Bias

Studies were quality-assessed using the Newcastle–Ottawa quality assessment scale (87% inter-rater agreement—the more stringent rating was chosen in cases of disagreement). Table 5 shows an overview grid of quality ratings, with rationales for decisions included in Supplementary Material. Mean study quality was 7/9 (“high quality” on average).

**Table 5:** Overview of Newcastle Ottawa quality assessment scale ratings

	Binnie (2016)	Holder (2019)	Lester (2010)	Mott (2014) (education variable)	Mott (2014) (income & employment variables)	Schindler (2012)
Selection						
Representativeness of exposed cohort	*	*	*	*	*	*
Non-exposed cohort selection	*	*	*	*	*	*
Ascertainment of exposure	*No explicit description*	*Written self-report*	*	*	*	*No description*
Outcome of interest not present at start of study	*	*	*	*	*	*
Compar-ability						
Comparability of cohorts—did study control for a key variable (1 point) and for any additional variable (1 point)	**	**	*	**	*Not for these variables*	*Not for deprivation variable*
Outcome						
Assessment of outcome	*	*	*No description*	*	*	*No description*
Follow-up long enough	*	*	*	*	*	*
Adequate follow-up	*	*	*	*	*	*
Total score (quality)	8/9 Very high	8/9 Very high	7/9 High	9/9 Very high	7/9 High	5/9 Moderate

Asterisks indicate scores of 1 (or 2), and Italic values indicate scores of 0 for the associated item. Mott (2014) appears twice, as it attained different scores for different deprivation variables

Two studies reported missing deprivation data (Lester et al., 2010; Mott et al., 2014; 20 and ≤ 10%, respectively). Sample size was relatively small in some studies, with < 50 dropouts reported by three of five studies. This limits power to detect effects. None of the five studies reported statements regarding conflicts of interest, risking bias via vested interests. The studies using trial data both reported a range of recruitment methods, reducing the chance of selection bias. Inclusion criteria for these studies were judged to be generally representative of routine practice.

Risk of publication bias was considered low, primarily because the variables of interest to this review were typically control variables in included studies. However, this may increase risk of outcome reporting bias, given deprivation analyses may not always be fully reported. Risk of selection bias was also considered low (see *Comparison with excluded studies*).

### Narrative Synthesis

An overview of statistical results is shown in Table 6. Associations between deprivation and dropout were tested using uncontrolled analyses (chi-square tests, t tests, or correlation matrices; 6 analyses) and/or controlled regression (5 analyses). Two out of six uncontrolled analyses found significant associations between deprivation and dropout, such that greater deprivation was associated with increased dropout (Binnie & Boden, 2016; Mott et al., 2014). The remaining four found no significant association. Furthermore, the significant results risk type I errors due to potential confounding from other variables.

**Table 6:** Statistical results from included studies

First author (year)	Measure of deprivation	Dropout definition	Summary results
Binnie (2016)	IMD	TJ	Uncontrolled chi-square analysis found that below average neighbourhood deprivation was significantly more common in completers (78%) compared with dropouts (64%). χ^2^(1) = 4.24, p = 0.039. However, logistic regression found that only depression severity remained significant as a dropout predictor (IMD was non-significant)
Holder (2019)	Education (years)	< 6 Sessions	Uncontrolled correlation matrix (p ≥ 0.05, r = 0.21) and logistic regression (p > 0.010, controlling for treatment outcome expectations and negative cognitions) both non-significant
		Sessions (out of 12) attended^a^	Uncontrolled correlation matrix (p ≥ 0.05, r = 0.23) and multiple regression (p > 0.010, controlling for treatment outcome expectations and negative cognitions) both non-significant
Lester (2010)	Education (years)	< Full protocol	Logistic regression (*B* = 0.81, OR 0.93, 95% CI 0.80–1.06, Z = − 1.08, p = 0.28) was non-significant after controlling for race, age, income, abuse history, treatment outcome expectations
Mott (2014)	Education (> high school)	TJ or < 7 sessions	Uncontrolled chi-square analysis (χ^2^(1) = 3.97, p < 0.05) found that post-high school education was significantly more common in completers (67%) compared with dropouts (45%). However, Logistic regression was non-significant after controlling for prior inpatient psychiatric stay and military service era
Schindler (2012)	Education (> 12 years)	TJ and < allowed sessions	Uncontrolled chi-square analysis (χ^2^(1) = 1.46, p > 1.00) was non-significant. Variable was therefore not entered into logistic regression
Lester (2010)	Household income	< Full protocol	Logistic regression (*B* = 0.68, OR 0.79, 95% CI 0.62–1.00, Z = − 2.00, p = 0.05) marginally reached significance after controlling for race, age, education, abuse history, treatment outcome expectations. Increased income was associated with reduced odds of dropout
Mott (2014)	Participant’s income	TJ or < 7 sessions	Uncontrolled t-test analysis (*t* = 0.75, p > 0.05) was non-significant. Variable was therefore not entered into logistic regression
Mott (2014)	Employment status	TJ or < 7 sessions	Uncontrolled chi-square analysis (χ^2^(1) = 1.46, p > 0.05) was non-significant. Variable was therefore not entered into logistic regression

Some studies appear more than once due to analysing more than one measure of deprivation

*IMD* index of multiple deprivation, *TJ* therapist judgement

^a^Continuous variable

After controlling for relevant variables (depression severity, prior inpatient stay, military service era) both of these significant effects became non-significant in logistic regression analyses. In total, only one out of five logistic regression analyses found that greater deprivation was significantly independently associated with increased dropout (Lester et al., 2010). The effect only marginally reached significance [*Z* score = 2.00, *OR* 1.27 (1.00–1.61), p = 0.05]. This effect was only significant for one of the two measures of deprivation analysed by that study (household income, but not education). The study did not control for symptom severity, which may have accounted for a proportion of variance apparently associated with deprivation, as in Binnie and Boden (2016). There was also 20% missing deprivation data reported, further limiting confidence. As such, on average the reviewed literature did not suggest a significant effect of deprivation on dropout.

The study reporting a significant controlled effect (Lester et al., 2010) had the largest sample size (over twice the average sample size of the other studies), and may have had more power to detect an effect, even accounting for missingness of data. It was the only study to analyse a household-level measure of deprivation, but otherwise was relatively similar to other included studies (a North American study of a cognitive processing therapy intervention targeting PTSD). Lester et al. (2010) defined dropout as not completing the full treatment protocol and post-treatment assessment. Because they do not describe the post-treatment assessment in detail, it is difficult to know whether this criterion may have affected dropout.

Three measures of deprivation were not included in studies’ logistic regression analyses, due to non-significance in uncontrolled analyses. Due to potential negative confounding effects, it cannot be assumed that these variables would have also been non-significant in a full logistic regression. Dropout rates and significance of effects did not appear to be systematically different for studies using RCT data compared with observational studies.

### Narrative Sub-group Comparisons

Because only one study found a significant independent (controlled) effect of deprivation, the scope for sub-group comparisons is limited. As such, although planned sub-group comparisons are presented in full in Supplementary Material, they are only briefly summarised here. Regarding *deprivation measure,* results from this review generally indicated no significant effect of education (*k* = 4). Other deprivation measures were reported by only 1–2 studies each. Regarding *delivery modality*, all included interventions were delivered face-to-face. Results may not therefore generalise to other modes of delivery. No clear patterns emerged between categories of *mental health disorder, study quality,* or *dropout measure*.

### Meta-Analysis

Controlled effects were preferred for the purposes of meta-analysis. Although only one study reported the required controlled effect size statistics (Lester et al., 2010), after contacting remaining authors by email, two further authors provided the required data (Binnie & Boden, 2016; Holder et al., 2019).

**Fig. 2:**
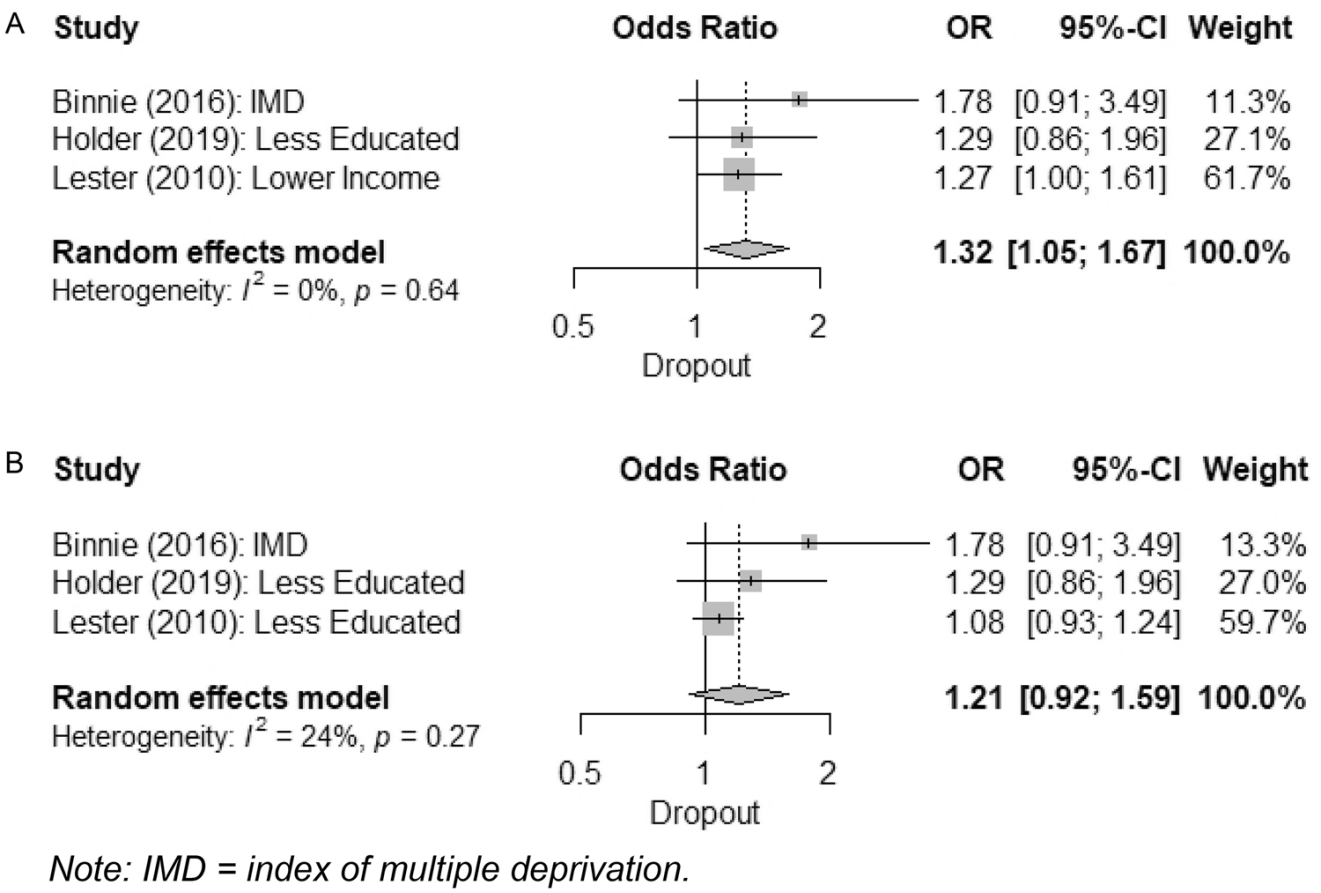
Meta-analyses of overall controlled effects of deprivation on dropout, using alternate measures of deprivation from Lester et al. (2010) in panel A and panel B

An overall meta-analysis of controlled effects of deprivation on dropout was performed using the three studies with relevant data (Binnie & Boden, 2016; Holder et al., 2019; Lester et al., 2010). As Lester et al. (2010) analysed both education and household income, two alternative analyses were performed (Fig. 2). Using household income in Lester et al. (2010), the overall effect was significant, such that greater deprivation was associated with increased odds of dropout, OR 1.32 (1.05–1.67), p = 0.019, with very low heterogeneity indicated by relevant tests, I^2^ = 0% (0–77%), Q = 0.89, p = 0.642. Substituting education in Lester et al. (2010), the overall effect was non-significant, OR 1.21 (0.92–1.59), p = 0.172. Heterogeneity was greater in this analysis, I^2^ = 24% (0–92%), Q = 2.62, p = 0.270, although both I^2^ confidence intervals were broad.

**Fig. 3:**
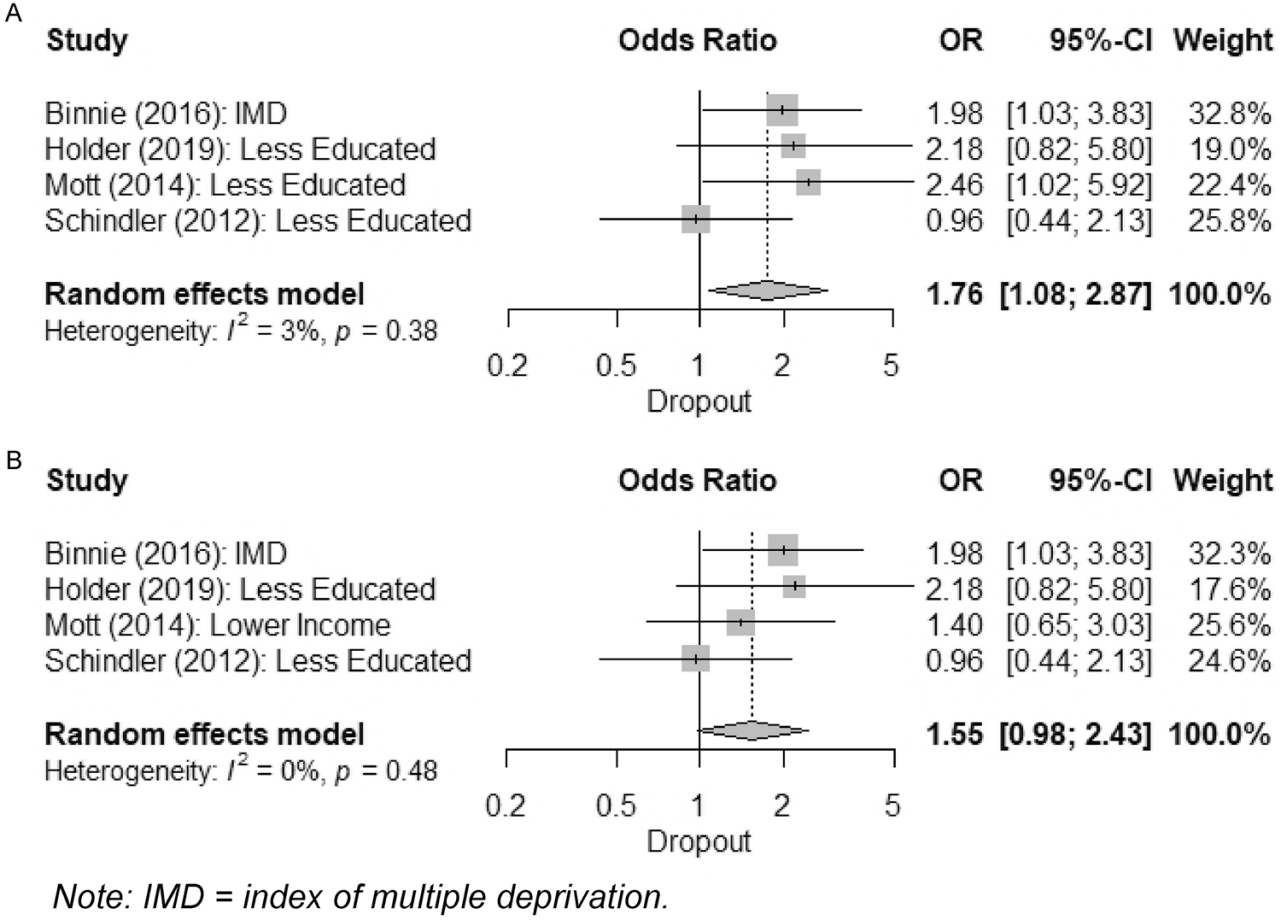
Meta-analyses of overall uncontrolled effects of deprivation on dropout, using alternate measures of deprivation from Mott et al. (2014) in panel A and panel B

Although controlled effect sizes were preferable, there were generally more studies reporting uncontrolled effect sizes, so meta-analyses of both are presented. An overall meta-analysis of uncontrolled effect of deprivation on dropout from four studies is presented in Fig. 3. Each of Mott et al.’s (2014) three measures of deprivation was meta-analysed separately. Using education (highest uncontrolled effect size) resulted in a significant overall uncontrolled effect of deprivation, such that greater deprivation was associated with increased odds of dropout, OR 1.76 (1.08–2.87), p = 0.024 (Fig. 3, Panel A). Tests indicated minimal heterogeneity, but broad confidence intervals [I^2^ = 3% (0–85%), Q = 3.09, p = 0.378].

Using income [lowest model heterogeneity, I^2^ = 0% (0–81%), Q = 2.45, p = 0.484] resulted in a non-significant overall uncontrolled effect, OR 1.55 (0.98–2.43), p = 0.059 (Fig. 3, Panel B). Similarly, using employment status [highest model heterogeneity, I^2^ = 49% (0–83%), Q = 5.91, p = 0.116] also resulted in a non-significant effect, OR 1.28 (0.70–2.34), p = 0.423 (not pictured). Because of the disparity in results﻿, a sensitivity analysis was conducted, excluding Mott et al. (2014). This was also non-significant, OR 1.60 (0.91–2.81), p = 0.105 (not pictured).

Thus, meta-analytic evidence for a general effect of deprivation (controlled or uncontrolled) is mixed. In both cases the effect crosses the threshold for significance depending on the measure of deprivation analysed by those studies using more than one measure. There does not appear to be a consistent trend (in particular, income but not education was significant in Lester et al., 2010 in the controlled analysis, and vice versa for Mott et al., 2014 in the uncontrolled analysis).

### Subgroup Meta-Analyses

As in the narrative synthesis, although subgroup meta-analyses and comparisons were planned, these were mostly unfeasible and/or uninterpretable due to low numbers of studies in each subgroup and risk of confounding. Only one subgroup (education) comprised more than 2 effects suitable for meta-analysis—this is therefore the only subgroup analysis tentatively reported here. Meta-analysis of the uncontrolled effect of education (*k* = 3) was non-significant, OR 1.67 (0.87–3.20), p = 0.121 [I^2^ = 30% (0–93%), Q = 2.87, p = 0.238].

### Comparison with Excluded Studies

Because of the low number of included studies, and the conservative inclusion criteria utilised, results from included studies were briefly compared with those from excluded studies. A comparative set of excluded studies was derived, including those studies that (a) were excluded during full-text assessment (i.e. passed initial screening), and (b) included an analysis of the effect of deprivation on dropout from individual psychological intervention for CMDs. In other words, although these studies still assessed the same broad topic, the stricter aspects of inclusion criteria were not enforced, giving a wider, more heterogeneous set of studies.

There were 15 studies in this comparator set (8 from database searching and 7 from citation searching; full list in Supplementary Material). Primary reasons for exclusion from the main review were: no clear exclusion of non-starters from the definition of dropout (*n* = 9), sample included a mix of both individual and group intervention participants (*n* = 4), or no clear differentiation between treatment dropout and study dropout (*n* = 2).

In summary, 6/14 uncontrolled analyses were significant, and 6/11 controlled analyses were significant. Three factors are briefly considered: reason for exclusion, deprivation measure, and target disorder.

Excluded comparator studies that may have included non-starters reported significant effects in 7/13 analyses (including 5/9 controlled analyses). Samples contaminated by group intervention participants reported significant effects in 4/6 analyses. Studies that did not differentiate treatment and study dropout reported significant effects in 0/3 analyses.

All 15 excluded comparator studies analysed the effect of education—7/15 found significant effects of education (including 4/9 controlled analyses). Two studies analysed the effect of income—both uncontrolled analyses were significant. Five studies analysed the effect of employment—2/5 were significant (including 2/2 controlled analyses). One study analysed the effect of socio-economic status—this uncontrolled analysis was non-significant.

As regards analyses in depression studies, 7/14 were significant. There were 3/5 significant effects in PTSD study analyses, and 1/4 significant effects in anxiety study analyses.

This comparison suggests that overall findings in the broader contemporary literature are also uncertain, although significant effects of deprivation may be more common compared with studies included in this review. Including non-starters and/or participants receiving group intervention may be associated with increased effects of deprivation, compared with the included literature that focused only on those who have already begun to attend treatment and are receiving individual treatment only. Inconclusive and tentative hints of differential effects according to deprivation measure or target disorder that were found in this review are also reflected in the broader literature.

## Discussion

This review aimed to assess the evidence for an effect of deprivation on dropout from contemporary psychological intervention for common mental disorders. Overall, evidence was inconclusive, based on five eligible studies. Narrative synthesis predominantly suggested no significant effect, especially after controlling for other covariates. Significance of meta-analyses varied according to the measure of deprivation in those studies that analysed multiple measures, and as such were uncertain.

Our findings contrast with older reviews (Baekeland & Lundwall, 1975; Garfield, 1994; Wierzbicki & Pekarik, 1993) that found mostly significant effects of variables such as socio-economic status, education, occupation, and income, but used broader dropout definitions and populations (for example, including people with severe mental illness and those receiving inpatient treatment).

In contrast, our results are more consistent with Swift and Greenberg’s (2012) more recent meta-regression, which found no significant effect of employment or education, despite also including broader clinical populations than the current review. One hypothesis is that changes to service provision or other factors over time have reduced the effect of deprivation on dropout, for example via initiatives to improve access to psychological interventions.

Research and review methods may also have improved over time. Wierzbicki and Pekarik (1993) note the potential for reporting bias to have inflated apparent effects, particularly in earlier studies. The relatively basic search terms and strategies used in earlier reviews (where reported) may also have led to selection bias. Both Baekeland and Lundwall (1975) and Garfield (1994) are particularly limited by current standards, with overwhelmingly opaque review methods, search strategies, and inclusion criteria. Garfield (1994) recognises that most studies in their review were of low quality. Wierzbicki and Pekarik’s (1993) approach to meta-analysis has also been challenged subsequently (Swift & Greenberg, 2012).

Furthermore, Baekeland and Lundwall’s (1975) review of adult outpatient psychotherapy studies found significant deprivation effects *only* for individually delivered psychoanalytic psychotherapy studies—no significant effects were detected for non-psychoanalytic studies (Baekeland & Lundwall, 1975). In contrast, the studies included in this review (and the majority of those in Swift & Greenberg, 2012) primarily used cognitive/cognitive-behavioural treatment orientations. This may indicate a moderating effect of treatment orientation—further investigation is warranted.

A relatively small effect of deprivation that is difficult to detect without suitable power is also a possibility. This interpretation is supported by the fact that sample size in this review was relatively small on average, particularly for participants who dropped out of treatment (around 50 on average per study). Consistent with this, the only significant controlled effect was detected by the study that had over double the average sample size of other studies (Lester et al., 2010). However, this contrasts with Swift and Greenberg’s (2012) meta-analysis, which was extremely highly powered. Explanations may relate to the broad range of clinical contexts they included, and/or to the measures of deprivation they did and did not analyse. In other words, there may also be differential effects. It is partly for this reason that carefully focused reviews are needed.

Deprivation itself is a multi-faceted concept, and proximal or distal indicators may demonstrate differing associations. Furthermore, a single measure may have differential effects under different treatment conditions. This was anticipated, and sub-group comparisons were planned and analysed where possible. However, the small number of studies and risk of study-level confounding made it difficult to draw firm conclusions. Education was most consistently analysed in the included studies—narrative and meta-analytic evidence from this review did not support a significant effect of education on dropout. This is consistent with Swift and Greenberg (2012), as well as a recent review and meta-analysis that found that education was a poorer predictor of clinical outcome than other deprivation measures (Finegan et al., 2018). Even Garfield (1994) reported inconsistent effects in their relatively more recent studies. Education was used in 50% of analyses in the current review, meaning our results may be biased towards this measure. It was not possible to draw meaningful conclusions regarding other specific measures of deprivation in the current review. It was also difficult to draw conclusions regarding target CMD, dropout definition, treatment delivery modality, or quality, in part due to risk of confounding.

This review aimed to use comprehensive search terms and inclusive supplementary search methods. As such, the low number of included studies was surprising. However, an earlier 15-year meta-analysis of dropout from individual psychotherapy (Sharf et al., 2010) also struggled to include eligible deprivation analyses, finding only one study providing usable effect size data (Sharf, 2009). The low number of included studies may be related to stringent inclusion criteria. These criteria were designed to produce relative homogeneity in the included study set, while limiting the potential for confounding by related concepts. In particular, differentiation was made in this review between treatment *non-initiation* and *dropout*. Failure to clarify this was the most common reason that studies otherwise meeting PICOSS criteria were excluded from the review. Including these would have tripled the number of included studies, but risked confounding with failure to initiate. Even then, a significant controlled effect would only have been found in 50% of studies. Tentative comparison of included and excluded studies suggests that it may be beneficial for future reviews to explore the potential for differential effects between non-initiators and dropouts, and/or between participants receiving group and individual intervention. There are indications from this review that conflating in-treatment dropout and non-initiation may potentially lead to inaccurate conclusions about deprivation and dropout.

Another potential impact of the highly selected set of studies included in the current review is the risk of selection bias, particularly regarding excluding internet-delivered interventions. For example, there were three internet-delivered intervention studies excluded primarily due to a failure to distinguish non-initiators from dropouts (although in practice their results were comparable to included studies). The concept of attending at least one session is arguably less intuitive for internet-delivered interventions, given they don’t typically involve sessions in the traditional sense. A comparable concept would be to specify that participants accessed the intervention at least once, or completed at least one module/chapter/video, and studies would have been included had they specified this. This could be specified in future studies, if only as a sensitivity analysis. Concepts such as attendance and dropout arguably begin to change meaning for some internet-based interventions, where therapist judgement may be inapplicable, and content may be accessed ad hoc according to patient need rather than scheduled between two parties. However, this also underscores the need for robust syntheses to compare and contrast effects across different modalities of delivery. Unfortunately, although it was an aim of this review, it was not possible in practice due to a lack of eligible studies.

Interestingly, a primary data meta-analysis with a narrow focus specifically on internet-based unguided cognitive behavioural therapy interventions for depression found that lower education level but not employment status predicted dropout (Karyotaki et al., 2015). Although dropout was again potentially confounded with treatment non-initiation, this could alternatively suggest that there may be a differential effect according to treatment modality. This is intuitive—for example, internet-based interventions may rely relatively more on literacy and information technology skills, increasing the salience of educational level (Waller & Gilbody, 2009). In comparison, face-to-face interventions require patients to travel to sessions, potentially requiring money for transport, child-care, etc. As such, factors such as income may be stronger predictors for face-to-face interventions.

One of the reasons for undertaking the present review was to attempt to capture emerging modalities such as internet and telephone based treatments. The failure to include studies in these areas is therefore troubling. Dropout is a major concern for internet treatments (from 10 unguided internet intervention RCTs, 40% of patients dropped out in the first quarter of treatment, with 70% dropout before completion of three quarters of modules; Karyotaki et al., 2015). It is therefore strongly recommended that future research into internet treatments in particular separates non-initiators from treatment dropouts, in order to improve understanding of what may be distinct processes.

Another contributory factor to the low number of included studies may be related to study design and reporting. Deprivation was not typically an explicit focus of the included and screened studies. Furthermore, typical measures of interest to this review were often included only as part of a range of control variables, and reported only in passing or in aggregate. This makes systematic assessment for inclusion particularly challenging. We were aware of this and aimed to err towards inclusivity when screening papers. Despite this, it is likely that some studies including relevant data were not identified. This is supported by the relatively high proportion of eligible studies identified through citation searching. The propensity for deprivation to be included only as a control variable also risks reporting bias. This was noted by Wierzbicki and Pekarik (1993), who found that up to 25% of demographic variable effect sizes could not be included in their meta-analysis due to insufficient reporting, and cautioning that their mean effects should be considered as upper bounds for true effects.

Another methodological limitation of the included literature was a failure to include deprivation measures in controlled analyses when they were non-significant in uncontrolled analyses. This increases the risk of type II error due to potential negative confounding in uncontrolled analyses, reduces the number of controlled analyses eligible for meta-analysis, and increases selection bias in the controlled analyses.

Similar to previous meta-analyses (Swift & Greenberg, 2012; Wierzbicki & Pekarik, 1993), there was variation in dropout rates across studies included in this review—in particular, four studies reported around 30–40% dropout whilst one reported 15%. Ironically, the outlier in our study was closest to Swift and Greenberg’s (2012) weighted mean dropout rate of 20%, whilst the remaining studies were comparable to Wierzbicki and Pekarik’s (1993) mean dropout rate of 47%. Both previous meta-analyses also reported wide variation in dropout (Swift & Greenberg range = 0–74%, Wierzbicki & Pekarik *SD* = 22%). Swift and Greenberg (2012) posit that these differences may reflect underlying covariates affecting dropout, although Wierzbicki and Pekarik (1993) also identified dropout operationalisation as a factor. The outlier study in this review used a relatively more conservative operationalisation of dropout, although the underlying components were consistent theoretically and practically with the broader dropout literature.

### Clinical Implications

Evidence from the current review is limited, with potential indications of differential effects despite overall negative or inconclusive findings. As such, clinic managers can neither assume nor rule out a contribution of deprivation to dropout from intervention, particularly as regards their specific clinical context. Results from this review also cannot be assumed to apply to treatment initiation—only to dropout subsequent to initiation.

Variables related to mental health severity and treatment expectations appeared to be stronger predictors of dropout than deprivation in controlled analyses. Care should be taken when interpreting these findings. In particular, causal relationships between these factors may be complex—for example, see social selection and social causation theories of mental health (Dohrenwend et al., 1992; Mossakowski, 2014). Treatment expectations may also be influenced by the individual’s socioeconomic position, with recent evidence indicating that therapeutic pessimism is greater for people experiencing deprivation (Potts & Henderson, 2020). Thus, although other variables may have better pragmatic predictive power for therapists and other front-line staff, policy makers should not yet discount deprivation as a potential contributory causal factor without more comprehensive evidence.

Clinics should also consider the choice of measures related to deprivation that they collect in routine clinical practice. Evidence suggests that education may be a weaker predictor of dropout and clinical outcome in contemporary face-to-face interventions, compared with other measures with more mixed predictive power. In addition to potentially differential effects according to delivery modality, effects may also vary by clinical context—clinics may benefit from conducting their own suitably powered context-specific evaluations, particularly as landscapes and policies regarding both clinical delivery and socio-economic inequality continue to evolve over time (e.g., Oates & Firth, 2020).

### Future Research Directions

Despite considerable literature investigating dropout covariates, there was only a very limited set of studies meeting inclusion criteria for this review. This was unexpected, and prevented us from fully utilising certain pre-planned analyses. We believe a picture may be emerging indicating differential effects of deprivation on dropout depending on context. As such a challenge for future reviewers may be how to balance homogeneity and applicability to current practice against sufficient included studies to make confident conclusions.

Future research (and practice) should continue to recognise that dropout may be defined in different ways, particularly as interventions evolve (for example, describing dropout from novel modalities and self-directed interventions). This can alter recorded rates of dropout and affect corresponding statistical results (Kaltenthaler et al., 2008; Richards & Richardson, 2012).

We recommend that deprivation variables included in analyses are clearly reported in titles and abstracts where possible. We also recommend that researchers clearly report their operationalisation of dropout, particularly regarding whether or not non-initiators are included as dropouts. Where they are included, a sensitivity analysis is strongly recommended with non-initiators analysed separated or excluded. Internet interventions might measure initiation by module views or access logs, etc. Similarly, individual and group intervention samples should be reportedly separately.

Studies that test deprivation measures in bivariate (uncontrolled) analyses should include them in multivariate analyses—again, if only as a secondary or sensitivity analysis. Open access data could also allow reviewers to more easily interrogate data to answer questions that were not asked at the time of the study. Results should be clearly reported (e.g., effect sizes, confidence intervals or standard errors and exact p values). Sample sizes should also be of sufficient power to detect potentially small effect sizes. Future research may benefit from looking beyond education to also consider other indicators of deprivation.

The American Psychological Association (APA) made recommendations for measuring markers of socioeconomic status almost fifteen years ago. Recommendations regarding education include measuring both the highest degree attained *and* years of education (American Psychological Association, 2007). The APA (2007) also suggest that wealth is a better measure than income at a specific point in time. They acknowledge that occupation can be more difficult to measure and that employment status as an alternative can provide useful information. Studies included in this review varied as regards concordance with these recommendations, most notably measuring income rather than wealth. Future studies (and clinical organisations) may benefit from considering these recommendations.

Contemporary studies designed directly to investigate the impact of deprivation are greatly needed. These studies could seek to differentiate between indicators of deprivation, as well as investigate potential causality and mediation effects with common predictors such as symptom severity and treatment expectations (Kling et al., 2007). Whilst a review specifically focused on the association between deprivation and non-initiation would also be warranted, any such review may encounter similar problems regarding sufficient contemporary studies. Finally, given our experience that deprivation variables are frequently not the main focus of studies and are only tangentially reported or understated, future reviews may consider hand-searching the full texts of a broader range of dropout studies (i.e. not searching specifically for deprivation-related terms), bearing in mind the need to factor in the greatly increased resource cost of such an approach.

## Conclusions

This review found that measures of deprivation tend not to predict dropout from contemporary face-to-face CMD interventions. However, the set of included studies was small, limiting confidence in these findings. More research is needed, and future studies need to clearly report both their definitions of dropout and their analyses of deprivation measures. Triangulation of evidence suggests that there may be differential effects according to clinical and methodological factors.

## Supplementary Information

Below is the link to the electronic supplementary material.

Supplementary file1 (DOCX 32 kb)

## Declarations

### Research Involving Human and Animal Participants

The article did not involve human participants or animals.
