# Supplementary material for: Socioeconomic Deprivation and Dropout from Contemporary Psychological Intervention for Common Mental Disorders: A Systematic Review
Source: Adm Policy Ment Health. 2021 Nov 27;49(3):490–505. doi: 10.1007/s10488-021-01178-8 (PMC9005422; doi:10.1007/s10488-021-01178-8)
Supplement: Supplementary file 1 — Supplementary file1 (DOCX 32 kb) [file 10488_2021_1178_MOESM1_ESM.docx]

***Supplementary Material***

[1 Example Search Strategy 2](#_Toc81408750)

[2 Data Collection Form 4](#_Toc81408751)

[3 Full quality assessment ratings 6](#_Toc81408752)

[4 Narrative sub-group comparisons 9](#_Toc81408753)

[5 Meta-analysis example code 11](#_Toc81408759)

[6 Studies in the Excluded Comparator Set 12](#_Toc81408760)

## Example Search Strategy

The following search strategy has been formatted to search Medline via the Ovid platform. This search strategy has been reformatted/translated to meet the requirements of each platform and/or database.

Ovid text searches used the *.mp* operator (where applicable, this searches in the title, abstract, original title, name of substance word, subject heading word, floating sub-heading word, keyword heading word, organism supplementary concept word, protocol supplementary concept word, rare disease supplementary concept word, unique identifier, synonyms). Web of Science searches used *topic* (title, abstract, author keywords, keywords plus). ProQuest searches used *NOFT* (anywhere except the full text). Cochrane searches used title, abstract, and keywords.

Limits: Year = 2010 onwards

| (psychology, clinical/ or community mental health services/ or psychotherapy/ or behavior therapy/ or cognitive behavioral therapy/ or "acceptance and commitment therapy"/ or mindfulness/ or desensitization, psychologic/ or eye movement desensitization reprocessing/ or dialectical behavior therapy/ or relaxation therapy/ or emotion-focused therapy/ or gestalt therapy/ or interpersonal psychotherapy/ or narrative therapy/ or person-centered psychotherapy/ or psychoanalytic therapy/ or psychotherapy, brief/ or psychotherapy, multiple/ or psychotherapy, psychodynamic/ or psychotherapy, rational-emotive/ or counseling/ or motivational interviewing/ or bibliotherapy/ or relaxation therapy/ |  |
| --- | --- |

OR

“acceptance and commitment” OR “acceptance & commitment” OR (behavio* activation) OR (behavio* intervention*) OR (behavio* therap*) OR (bibliotherap*) OR (cognitive analytic therap*) OR (cognitive analytic treatment*) OR (cognitive analytic intervention*) OR (CBASP) OR (Cognitive Behavioral Analysis System of Psychotherapy) OR (CBT) OR (cognitive behavio* therap*) OR (cognitive behavio* intervention*) OR (cognitive behavio* treatment*) OR (cognitive ADJ2 intervention*) OR (cognitive ADJ2 treatment*) OR (cognitive ADJ2 therap*) OR (CFT) OR (compassion focus?ed therap*) OR (compassion focus?ed treatment*) OR (compassion focus?ed intervention*) OR (DBT) OR (dialectical behavio* therap*) OR (dialectical behavio* treatment*) OR (dialectical behavio* intervention*) OR (EMDR) OR (eye movement desensiti#ation) OR (exposure therap*) OR (exposure treatment*) OR (exposure intervention*) OR (Functional Analytic Psychotherap*) OR (guided self help) OR (guided self-help) OR (hypnotherapy*) OR (MBCT) OR (MBSR) OR (mindfulness based cognitive therap*) OR (mindfulness based cognitive treatment*) OR (mindfulness based cognitive intervention*) OR (mindfulness based stress reduction) OR (mindfulness ADJ2 therap*) OR (mindfulness ADJ2 treatment*) OR (mindfulness ADJ2 intervention*) OR (metacognitive therap*) OR (metacognitive treatment*) OR (metacognitive intervention*) OR (motivational interview*) OR (problem solving therap*) OR (problem solving treatment*) OR (problem solving intervention*) OR (psych* therap*) OR (psych* treatment*) OR (psych* intervention*) OR (psychotherap*) OR (psychoanalytic therap*) OR (psychoanalytic treatment*) OR (psychoanalytic intervention*) OR (psychodynamic therap*) OR (psychodynamic treatment*) OR (psychodynamic intervention*) OR (psychoeducat*) OR (psycho* management) OR (relaxation technique*) OR (relaxation therap*) OR (relaxation treatment*) OR (relaxation intervention*) OR (stress inoculation) OR (supportive therap*) OR (Emotion focused therap*) OR (emotion-focused therap*) OR (gestalt therap*) OR (narrative therap*))

AND

(Working Poor/ or Poverty/ or socioeconomic factors/ or economic status/ or poverty areas/ or social class/ or hierarchy, social/ or income/ or educational status/ or academic failure/ or literacy/ or employment/ or unemployment/

OR

(poor* area*) OR (poor* communit*) OR (poor* people*) OR (poor* patients) OR (poor* neighbo?rhood*) OR (poverty) OR (impover*) OR (social class*) OR (social status*) OR (social rank*) OR (social group*) OR (socioeconomic*) OR (socio-economic*) OR (SES) OR (SEP) OR (economic status*) OR (depriv* not (androgen depriv* or sleep depriv* or sensory depriv* or oxygen depriv* or glucose depriv* or nutrient depriv*)) OR (financial hardship) OR (financial* distress*) OR (financial difficulty) OR (financial* burden*) OR (financial* disadvanta*) OR (income) OR (wage) OR (low* earn*) OR (low* salar*) OR (under?employ*) OR (under employ*) OR (un?employ*) OR (non?employ*) OR (education* depriv*) OR (education* level*) OR (education* attain*) OR (education* disadvantage*) OR (education* status) OR (occupation* status) OR (disadvantag* communit*) OR (disadvantag* neighbo?rhood*) OR (disadvantag* area*) OR (disadvantag* patient*) OR (food insecurity) OR (debt*) OR (destitut*))

AND

("treatment adherence and compliance"/ or patient dropouts/ or treatment refusal/ or No-Show Patients/

OR

(attend) OR (attendance) OR (attended) OR (drop out) OR (dropout) OR (drop-out) OR (dropping out) OR (non?attend*) OR (non attend*) OR (non?adher*) OR (non adher*) OR (fail* ADJ2 attend*) OR (miss* ADJ1 appointment*) OR (miss* ADJ1 session*) OR (unilateral terminati*) OR (unilateral withdraw*) OR (unilateral discontinu*) OR (no show) OR (no-show) OR (failed appointment*) OR (show rate*) OR (appointment ADJ2 adher*) OR (session ADJ2 adher*) OR (attrition) OR (treatment* ADJ2 retention) OR (service* ADJ2 retention) OR (care ADJ2 retention) OR (therap* ADJ2 retention) OR (intervention* ADJ2 retention) OR (clinic* ADJ2 retention) OR (treatment* ADJ2 adher*) OR (service* ADJ2 adher*) OR (care ADJ2 adher*) OR (therap* ADJ2 adher*) OR (intervention* ADJ2 adher*) OR (clinic* ADJ2 adher*) OR (treatment* ADJ2 withdraw*) OR (service* ADJ2 withdraw*) OR (care ADJ2 withdraw*) OR (therap* ADJ2 withdraw*) OR (intervention* ADJ2 withdraw*) OR (clinic* ADJ2 withdraw*) OR (treatment* ADJ2 refus*) OR (service* ADJ2 refus*) OR (care ADJ2 refus*) OR (therap* ADJ2 refus*) OR (intervention* ADJ2 refus*) OR (treatment* ADJ2 discontinu*) OR (service* ADJ2 discontinu*) OR (care ADJ2 discontinu*) OR (therap* ADJ2 discontinu*) OR (intervention* ADJ2 discontinu*) OR (clinic* ADJ2 discontinu*) OR (treatment* ADJ2 terminat*) OR (service* ADJ2 terminat*) OR (care ADJ2 terminat*) OR (therap* ADJ2 terminat*) OR (intervention* ADJ2 terminat*) OR (treatment* ADJ2 disengag*) OR (service* ADJ2 disengag*) OR (care ADJ2 disengag*) OR (therap* ADJ2 disengag*) OR (intervention* ADJ2 disengag*) OR (clinic* ADJ2 disengag*))

## Data Collection Form

Data was collected using a Microsoft Excel database. A specification of this database is shown below.

The following data was extracted from all screened records:

- Study ID
- Reference data (authors, year of publication, title, journal, etc.)
- Stage at which decision to exclude is made (screening / full-text / not excluded)
- Exclusion reason(s) (with location in text)
  - Study type
  - Participants
  - Intervention
  - Comparison
  - Outcome
- Notes

In addition, the following data was extracted from all included records (along with corresponding locations in text):

- **General information**
  - Date of extraction
  - Contact email address
  - Research design
  - Research question
  - Key conclusions of authors
  - Ethical approval needed/reported (yes, no, n/a, unclear)
  - Funding source & role of funders
  - Possible conflicts of interest
- **Population & Setting**
  - Population description
  - Setting description
  - Country
  - Inclusion & exclusion criteria
  - Recruitment, sampling, randomisation, & withdrawal/exclusion procedures
  - Baseline imbalances
  - Sample size (per group/subsample if relevant)
  - Sample characteristics: (mean, SD, range for continuous variables, proportions for categorical variables, or alternate statistics if others are provided) – Age, Gender/Sex, Ethnicity/Race, Employment status, relevant socio-demographics
  - Mental health disorder(s), and severity, chronicity, comorbidities
- **Subsamples/intervention groups**
  - Group name
  - Group size (N)
  - Description
  - Intervention(s) description
  - Delivery medium (e.g. face to face, telephone, etc.)
  - Intervention duration – sessions (mean, range, SD, other data)
  - Intervention duration - time (mean, range, SD, other data)
  - Intervention target disorder/symptoms
  - Providers / therapists
  - Co-interventions
  - Notes
- **Comparators**
  - Comparator name
  - Comparator definition
  - Type of deprivation
  - Comparator level (e.g. individual, neighbourhood)
  - Type of variable (categorical, continuous, etc.)
  - Comparator operationalisation (incl. scale/categories)
  - Descriptive statistics
  - Validated measure?
  - Source of comparator data
  - Time points measured/reported
  - Handling of missing data
  - Notes
- **Outcomes**
  - Outcome name
  - Outcome definition
  - Outcome operationalisation
  - Descriptive statistics
  - Person measuring/reporting outcome
  - Time points measured
  - Time points reported
  - Handling of missing data
  - Power
  - Notes
- **Risk of bias/Quality assessment**
  - Risk of bias assessed by the Newcastle-Ottawa Quality Assessment Scale.
- **Data and analysis**
  - Comparison
  - Outcome
  - Subgroup
  - Unit of analysis
  - Results
    - Dichotomous outcome – no. with event/total in group, by group
    - Continuous outcome – mean & SD, by group
    - Other outcome – results, by group
  - Any other statistical results (raw differences, effect sizes, CI, p values, as provided in study manuscripts)
  - No. missing participants
  - Reasons missing
  - Statistical methods and appropriateness
  - Notes
- **Applicability**
  - Have important populations been excluded from the study?
  - Is the intervention likely to be aimed at disadvantaged/deprived groups?
  - Does the study directly address the review question?
  - Notes

## Full quality assessment ratings

Note: Green values indicate scores of 1, and white values indicate scores of 0 for the associated item. Mott (2014) appears twice, as it attained different scores for different deprivation variables.

|  |  | Binnie (2016) | Holder (2019) | Lester (2010) | Mott (2014) (education variable) | Mott (2014) (income & employment) | Schindler  (2012) |
| --- | --- | --- | --- | --- | --- | --- | --- |
| Selection | Representativeness of exposed cohort | a/b) somewhat to truly representative - the sample is not a selected group. The sample was somewhat skewed towards lower deprivation (e.g. <50% above average IMD), however this is likely to reflect the actual population accessing services. | a/b) somewhat to truly representative - inclusion/exclusion criteria appear reasonable compared with routine practice, although males were excluded from the sample (for good reason in this study, as all males completed treatment) | a/b) somewhat to truly representative - different inclusion criteria between studies, e.g. one study required completed rape, whilst the other required sexual or physical assault. However, inclusion criteria broadly reflected what might be assumed in routine practice. | a/b) somewhat to truly representative – routine practice based full sampling was used, but only for 8 out of 9 therapists (one therapist declined to participate) - although selection was on the therapist, this may have biased the sample - it is difficult to be confident without more information. | See left. | a) truly representative - no indications of non-representativeness for the population in question |
|  | Non-exposed cohort selection | a) drawn from the same community – single selection of both exposed and non-exposed cohorts | a) drawn from the same community – single selection of both exposed and non-exposed cohorts | a) drawn from the same community – single selection of both exposed and non-exposed cohorts | a) drawn from the same community – single selection of both exposed and non-exposed cohorts | See left | a) drawn from the same community – single selection of both exposed and non-exposed cohorts |
|  |  | Binnie (2016) | Holder (2019) | Lester (2010) | Mott (2014) (education) | Mott (2014) (income & employment) | Schindler  (2012) |
| Selection | Ascertainment of exposure | d) no description - secure record is strongly implied as iaptus is identified as a primary data source, and IMD is calculated from postcode, which should be recorded on iaptus, but this is not explicitly specified. | c) written self-report | b) structured interview | a) secure record - chart review "To obtain more detailed information on patients’ characteristics and use of psychotherapy, we reviewed the 91 medical records of EBP initiators". Chart review is later described, and demographics are reported for the "chart review sample" | See left | d) no description |
|  | Outcome of interest not present at start of study | a) yes - e.g. the text states that patients attended at least 1 session (p238), other types of ending were excluded from analysis | a) yes - patients attended at least one individual outpatient psychotherapy session (p89) | a) yes - e.g. Table 1, distinguishing "never started" from dropouts | a) yes - patients attended at least one individual outpatient psychotherapy session (p266) | See left | a) yes - patients attended at least one treatment session (p366) |
| Comparability | Comparability of cohorts - did study control for a key variable (1 point) and for any additional variable (1 point) | a) & b) study controls for pre-treatment mental health symptom severity and level of clinical risk | a) & b) study controls for age, depressive symptoms, PTSD symptoms, therapy expectations, negative cognitions | b) study controls for abuse history, treatment expectations, age, race, and two forms of deprivation (but doesn't control for symptom severity) | a) & b) study controls for a broad range of variables, including e.g. psychiatric inpatient history, psychiatric diagnoses, psycho-social functioning, suicide risk, PTSD service connection. | c) These two deprivation variables (income and employment) were not tested into the logistic regression - only bivariate analyses. | c) no control variables used in analysis of deprivation variable (although they were in the full logistic regression, the deprivation measure was not included in that analysis, only in bivariate analysis). |
|  |  | Binnie (2016) | Holder (2019) | Lester (2010) | Mott (2014) (education) | Mott (2014) (income & employment) | Schindler  (2012) |
| Outcome | Assessment of outcome | b) record linkage | b) record linkage | d) no description | b) record linkage - completion status defined "by chart review" | See left | d) no description |
|  | Follow-up long enough | a) yes - all patients included in analysis had been discharged (either completed or dropped out) | a) yes - all patients included in analysis had been discharged (either completed or dropped out) | a) yes - all patients included in analysis had been discharged (either completed or dropped out) | a) yes - all patients included in analysis had been discharged (either completed or dropped out) | See left | a) yes - all patients included in analysis had been discharged (either completed or dropped out) |
|  | Adequate follow-up | a) complete follow up - all participants accounted for | a) complete follow up - all participants accounted for | a) complete follow up - all subjects accounted for | a) complete follow up - all subjects accounted for | See left | a) complete follow up - all subjects accounted for |
| Selection Score | | 3/4 | 3/4 | 4/4 | 4/4 | 4/4 | 3/4 |
| Comparability Score | | 2/2 | 2/2 | 1/2 | 2/2 | 0/2 | 0/2 |
| Outcome Score | | 3/3 | 3/3 | 2/3 | 3/3 | 3/3 | 2/3 |
| **Total Score** | | **8/9** | **8/9** | **7/9** | **9/9** | **7/9** | **5/9** |
| Note: Green values indicate scores of 1, and white values indicate scores of 0 for the associated item. Mott (2014) appears twice, as it attained different scores for different deprivation variables. | | | | | | | |

## Narrative sub-group comparisons

## Delivery modality

Although this review planned to compare studies on the basis of delivery modality, this was not possible because all included interventions were delivered face-to-face. Results may not therefore generalise to other modes of delivery such as telephone or web-based interventions.

## Dropout measure

Some form of significant effect of deprivation was found in at least one study using each approach to measuring dropout (duration-based, *k* = 2; therapist judgement; *k* = 1; combination, *k* = 2). However, the only significant controlled effect used a duration based measure. These results are inconclusive. One study used a continuous definition of dropout, in addition to their binary definition (Holder et al., 2019). All results in this study were non-significant.

## Deprivation measure

***Education*.** Four studies analysed the effect of education on dropout. Results from two of three uncontrolled analyses, all three logistic regressions, and a further sensitivity analysis (continuous outcome) were all non-significant. These results suggest that education is not a significant predictor of dropout.

***Income*.** Two North American studies focusing on CPT interventions for PTSD analysed the effect of income on dropout. Lester et al.’s (2010) large study of female victims of violence found a marginal significant effect of household income, whilst Mott et al.’s (2014) relatively small study of predominantly male veterans did not find an effect of individual income. Although gender was a distinguishing factor in these studies (see also Holder et al., 2019), differential effects cannot be assumed without further evidence. Similar caution should be taken when considering any differential impact of household vs individual income, although it is arguably intuitive that household income may provide a better indicator of deprivation for people who have a shared household income.

***Other Measures*.** Only one study each assessed neighbourhood deprivation and employment status. Relative neighbourhood deprivation (above/below the average in England) exhibited an uncontrolled association with dropout that became non-significant after controlling for depression severity (Binnie & Boden, 2016). Employment status was not significant in an uncontrolled (chi-square) analysis (Mott et al., 2014). This was the only measure not included in a controlled analysis, increasing chances of type II error due to negative confounding, particularly given the relatively small sample.

## Mental health disorder

Three studies targeted PTSD (Holder et al., 2019; Lester et al., 2010; Mott et al., 2014), one study targeted depression (Schindler et al., 2013), and one targeted a variety of CMDs (Binnie & Boden, 2016). Due to risk of confounding, mental health disorder was considered to be less reliable as a predictor, compared with other potential factors. For example, Schindler et al. (2013) was the only study to investigate depression, but their non-significant findings are considered more likely to be due to the measure of deprivation used (education), and being the only German study means that cultural/socio-political factors cannot be ruled out. Similarly, Binnie and Boden (2016) was the only study not to focus on a specific CMD, the only British study, and the only one to investigate neighbourhood deprivation. The three studies targeting PTSD were all North American studies, with varied but generally non-significant findings. In summary, the narrative evidence for any one specific CMD is considered inconclusive.

## Study quality

All three very high quality (controlled) analyses were non-significant. Results from high quality analyses were mixed, whilst the only moderate quality (uncontrolled) analysis did not detect an effect (Schindler et al., 2013).

## Meta-analysis example code

#overall meta-analysis

#lower and upper CIs are also log-transformed

#example overall controlled effect data below

#author logOR lower upper

#Binnie (2016): IMD 0.577736329 -0.095410185 1.250760966

#Holder (2019): Less Educated 0.257738 -0.23785118 0.587608871

##Lester (2010): Lower Income 0.235722334 0 0.478033801

##Lester (2010): Less Educated 0.072570693 -0.058264908 0.223143551

study_data$seTE <- (study_data$upper - study_data$lower) / 3.92

authors <-study_data$author

#display results

meta::metagen(logOR,seTE,studlab = authors,

method.tau = "SJ",

sm = "OR",

data = study_data)

#output results to object for use in forest plot

metaresults <-meta::metagen(logOR,seTE,studlab = authors,

method.tau = "SJ",

sm = "OR",

data = study_data)

#display forest plot

meta::forest.meta(metaresults, comb.fixed = FALSE, xlab = "Dropout", leftcols = c("studlab"), print.tau2 = FALSE)

#moderator meta analysis

#lower and upper CIs are also log-transformed

# example PTSD moderator data below

#author logOR lower upper

#Binnie (2016): IMD 0.684862961 0.027323296 1.34231635 non-PTSD

#Holder (2019): Less Educated 0.779324877 -0.198450939 1.759580571 PTSD

#Mott (2014): Less Educated 0.901461318 0.023814178 1.779062537 PTSD

#Schindler (2012): Less Educated -0.037494204 -0.83171983 0.756685201 non-PTSD

study_data$seTE <- (study_data$upper - study_data$lower) / 3.92

authors <-study_data$author

meta::metagen(logOR,seTE,studlab = authors,

method.tau = "SJ",

sm = "OR",

data = study_data, byvar = study_data$subgroup)

metaresults <-meta::metagen(logOR,seTE,studlab = authors,

method.tau = "SJ",

sm = "OR",

data = study_data, byvar = study_data$subgroup)

meta::forest.meta(metaresults, comb.fixed = FALSE, xlab = "Dropout", leftcols = c("studlab"), print.tau2 = FALSE, print.I2 = FALSE)

## Studies in the Excluded Comparator Set

Berke, D. S., Kline, N. K., Wachen, J. S., McLean, C. P., Yarvis, J. S., Mintz, J., Young-McCaughan, S., Petersong, A. L., Foa, E., Resick, P. A., Litz, B. T., & Consortium, S. S. (2019). Predictors of attendance and dropout in three randomized controlled trials of PTSD treatment for active duty service members. *Behaviour Research and Therapy*, *118*, 7-17. https://doi.org/10.1016/j.brat.2019.03.003

Bisseling, E., Cillessen, L., Spinhoven, P., Schellekens, M., Compen, F., van der Lee, M., & Speckens, A. (2019). Development of the Therapeutic Alliance and its Association With Internet-Based Mindfulness-Based Cognitive Therapy for Distressed Cancer Patients: Secondary Analysis of a Multicenter Randomized Controlled Trial. *Journal of Medical Internet Research*, *21*(10), Article e14065. https://doi.org/10.2196/14065

Blanco, C., Markowitz, J. C., Hellerstein, D. J., Nezu, A. M., Wall, M., Olfson, M., Chen, Y., Levenson, J., Onishi, M., Varona, C., Okuda, M., & Hershman, D. L. (2019). A randomized trial of interpersonal psychotherapy, problem solving therapy, and supportive therapy for major depressive disorder in women with breast cancer. *Breast Cancer Research and Treatment*, *173*(2), 353-364. https://doi.org/10.1007/s10549-018-4994-5

Cook, J. M., Thompson, R., Harb, G. C., & Ross, R. J. (2013). Cognitive-Behavioral Treatment for Posttraumatic Nightmares: An Investigation of Predictors of Dropout and Outcome. *Psychological Trauma-Theory Research Practice and Policy*, *5*(6), 545-553. https://doi.org/10.1037/a0030724

El Alaoui, S., Ljotsson, B., Hedman, E., Kaldo, V., Andersson, E., Ruck, C., Andersson, G., & Lindefors, N. (2015). Predictors of Symptomatic Change and Adherence in Internet-Based Cognitive Behaviour Therapy for Social Anxiety Disorder in Routine Psychiatric Care. *Plos One*, *10*(4), Article e0124258. https://doi.org/10.1371/journal.pone.0124258

Hoyer, J., Wiltink, J., Hiller, W., Miller, R., Salzer, S., Sarnowsky, S., Stangier, U., Strauss, B., Willutzki, U., & Leibing, E. (2016). Baseline Patient Characteristics Predicting Outcome and Attrition in Cognitive Therapy for Social Phobia: Results from a Large Multicentre Trial. *Clinical Psychology & Psychotherapy*, *23*(1), 35-46. https://doi.org/10.1002/cpp.1936

Hungerbuehler, I., Valiengo, L., Loch, A. A., Rossler, W., & Gattaz, W. F. (2016). Home-Based Psychiatric Outpatient Care Through Videoconferencing for Depression: A Randomized Controlled Follow-Up Trial. *Jmir Mental Health*, *3*(3), Article UNSP e36. https://doi.org/10.2196/mental.5675

Jarrett, R. B., Minhajuddin, A., Kangas, J. L., Friedman, E. S., Callan, J. A., & Thase, M. E. (2013). Acute phase cognitive therapy for recurrent major depressive disorder: Who drops out and how much do patient skills influence response? *Behaviour Research and Therapy*, *51*(4-5), 221-230. https://doi.org/10.1016/j.brat.2013.01.006

Keefe, J. R., Chambless, D. L., Barber, J. P., & Milrod, B. L. (2020). Predictors and moderators of treatment dropout in cognitive-behavioral and psychodynamic therapies for panic disorder. *Psychotherapy Research*. https://doi.org/10.1080/10503307.2020.1784487

Keefe, J. R., Stirman, S. W., Cohen, Z. D., DeRubeis, R. J., Smith, B. N., & Resick, P. A. (2018). In rape trauma PTSD, patient characteristics indicate which trauma-focused treatment they are most likely to complete. *Depression and Anxiety*, *35*(4), 330-338. https://doi.org/10.1002/da.22731

Lopes, R. T., Goncalves, M. M., Sinai, D., & Machado, P. P. P. (2015). Predictors of dropout in a controlled clinical trial of psychotherapy for moderate depression. *International Journal of Clinical and Health Psychology*, *15*(1), 76-80. https://doi.org/10.1016/j.ijchp.2014.11.001

Maguen, S., Li, Y. M., Madden, E., Seal, K. H., Neylan, T. C., Patterson, O. V., DuVall, S. L., Lujan, C., & Shiner, B. (2019). Factors associated with completing evidence-based psychotherapy for PTSD among veterans in a national healthcare system. *Psychiatry Research*, *274*, 112-128. https://doi.org/10.1016/j.psychres.2019.02.027

Schmidt, I. D., Forand, N. R., & Strunk, D. R. (2019). Predictors of Dropout in Internet-Based Cognitive Behavioral Therapy for Depression. *Cognitive Therapy and Research*, *43*(3), 620-630. https://doi.org/10.1007/s10608-018-9979-5

Vohringer, M., Knaevelsrud, C., Wagner, B., Slott, M., Schmidt, A., Stammel, N., & Bottche, M. (2020). Should I stay or must I go? Predictors of dropout in an internet-based psychotherapy programme for posttraumatic stress disorder in Arabic. *European Journal of Psychotraumatology*, *11*(1), Article 1706297. https://doi.org/10.1080/20008198.2019.1706297

Zeng, Q., He, Y., Shi, Z., Liu, W., Tao, H., Bu, S., Miao, D., Liu, P., Zhang, X., Li, X., Qi, X., & Zhou, Q. (2016). A community-based controlled trial of a comprehensive psychological intervention for community residents with diabetes or hypertension. *Shanghai archives of psychiatry*, *28*(2), 72-85. https://doi.org/10.11919/j.issn.1002-0829.216016
